# The MRI-Visible Nanocomposite Facilitates the Delivery and Tracking of siRNA Loaded DC Vaccine in the Breast Cancer Model

**DOI:** 10.3389/fonc.2020.621642

**Published:** 2021-02-05

**Authors:** Changqiang Wu, Wencheng Zhu, Rongrong Jin, Hua Ai, Ye Xu

**Affiliations:** ^1^ Department of Radiology, Children’s Hospital of Chongqing Medical University, National Clinical Research Center for Child Health and Disorders, Ministry of Education Key Laboratory of Child Development and Disorders, Chongqing Key Laboratory of Pediatrics, Chongqing, China; ^2^ Sichuan Key Laboratory of Medical Imaging and School of Medical Imaging, North Sichuan Medical College, Nanchong, China; ^3^ National Engineering Research Center for Biomaterials Sichuan University, Chengdu, China; ^4^ Shanghai Institute of Biochemistry and Cell Biology, Chinese Academy of Sciences, Shanghai, China

**Keywords:** dendritic cell, anticancer immunotherapy, gene transfection, nanocomposite, magnetic resonance imaging

## Abstract

Dendritic cell (DC) vaccines have recently been developed for the treatment of various cancers but often do not function as well as expected, primarily due to the highly complex *in vivo* immune environment. This proof-of-principle study aimed to test the feasibility of modulating the *in vivo* behaviors of DC vaccines (DCVs) by introducing siRNA-laden magnetic resonance (MR) imaging nanovectors into cells, while providing visible information on their homing to lymph nodes. The N-alkyl-PEI2k-LAC/SPIO nanocomposites were prepared and characterized, showing favorable properties of siRNA transfection and MRI labeling efficiency in DCs. Cell viability assays revealed no observable effects on the survival and phenotype of DCs if the concentration of the complex was within 8 μg Fe/ml. An orthotopic mouse model of breast cancer was developed. The DCVs transfected with *IDO* siRNA contained nanocomposites were adoptively transferred to start the treatment. MR imaging clearly visualized the homing of DCVs into lymph nodes. At the end of the treatment, DCVs presented significantly better tumor suppression than DCs or PBS (P < 0.05). Generally, the N-alkyl-PEI2k-LAC/SPIO nanocomposites represent a highly efficient MR imaging platform for siRNA transfection that is potentially useful for *in vivo* tracking of vaccine cells.

## Introduction

**Figure 1:**
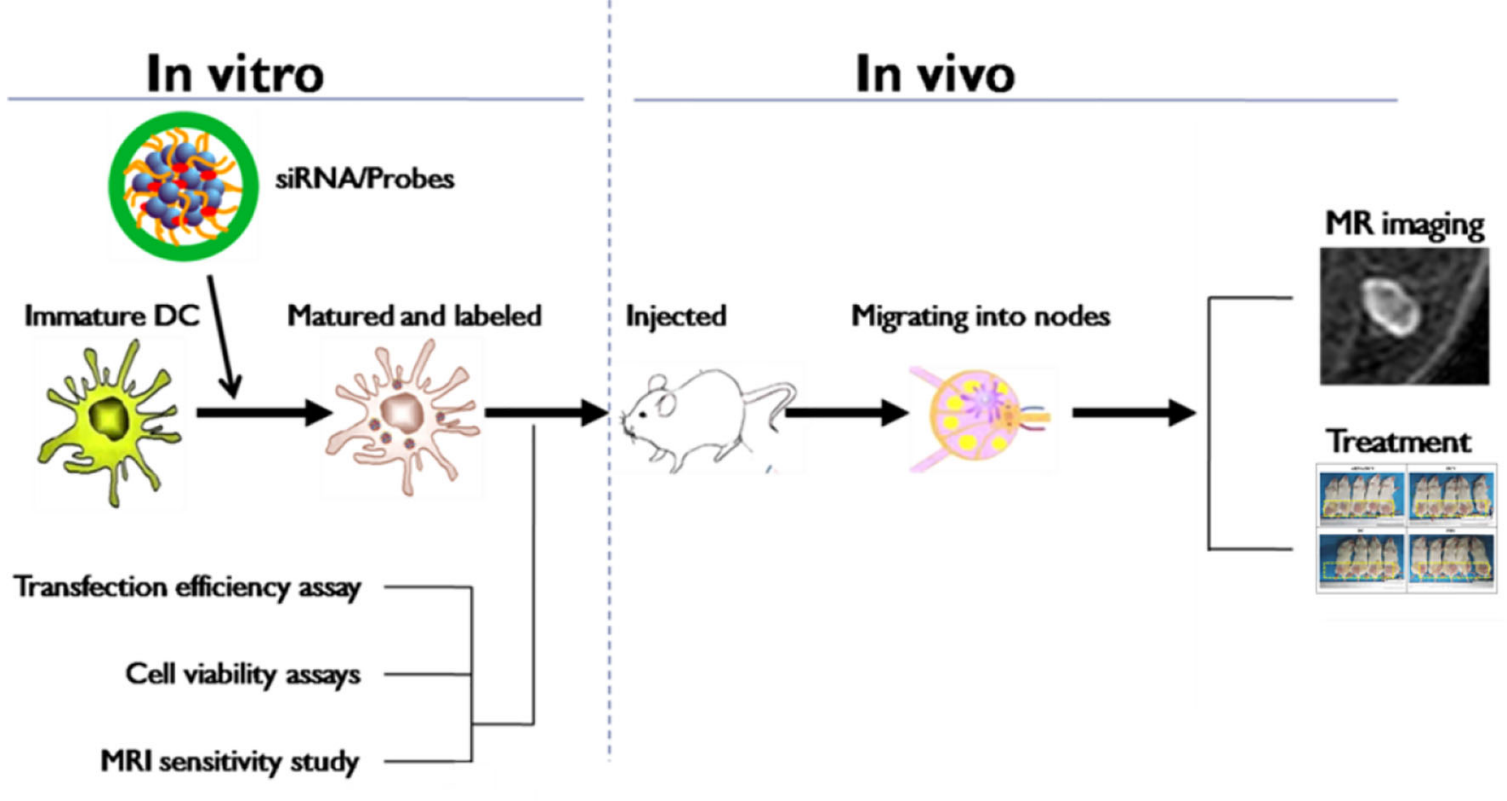
Schematic of the experimental procedures.

Dendritic cells (DCs) have been recruited as a cellular vaccine for tumor immunotherapy but their performance *in vivo* is generally unsatisfactory (1). Factors such as the weak antigenicity of tumors, failure of vaccine cells to migrate into lymph nodes (LNs) and drive T cell priming, and immune evasion of tumors, *etc.*, are considered the culprits (2). Among these factors, tumor immune evasion may play a key role. Recent studies revealed that up-regulated expression of indoleamine 2, 3-dioxygenase (IDO), a major rate-limiting enzyme of tryptophan catabolism, in DCs would induce naïve CD4^+^ T cells to differentiate into regulatory T cells (T-regs), thus impeding antitumor immunity (3). A small interfering RNA (siRNA) targeting IDO has been transfected into DCs to block the immunosuppression induced by the *IDO* mRNA, which exhibited potent tumor growth inhibition in animal models (4–6). As a promising RNA interference (RNAi) strategy, it involves the processes of delivering synthetic siRNAs into cells, incorporating these siRNAs into the silencing complex and subsequent degradation of sequence-specific mRNA. Unfortunately, exogenous siRNAs are easily degraded by nuclease *in vivo*, restricting the application of RNAi. Therefore, the preparation of an effective gene delivery system for loading and protecting gene fragments from nuclease damage and for improving gene transfection efficiency is very important (7). Previously, our research group synthesized an aqueous-phase magnetic resonance (MR) nanovector, N-alkyl-PEI2k/SPIO, with superior properties in subsequent experiments for MR imaging and as a nanocarrier for gene transfection (8, 9). Moreover, because the nanocomposites are modified by lactose, they hold superior MR imaging properties, improved biocompatibility by inducing protective autophagy and enhanced therapeutic immune activation of DC (10, 11). This study aimed to employ this lactosylated analog as a gene delivery platform for carrying *IDO* siRNA to transfect antitumor DC vaccines (DCVs) and to use it for the treatment of orthotopic 4T1 breast cancer in mouse models while using MR imaging to track the *in vivo* homing of DCVs to the draining LNs (**Figure 1**). To our best knowledge, this study utilizes a self-made, MRI-visible gene transfection vector to modulate the anticancer functions of DCVs by gene silencing while tracking their migration *in vivo*. Few studies have been reported on this topic previously.

## Materials and Methods

### Preparation of N-alkyl-PEI2k-LAC-Stabilized SPIO Nanoparticles

Branched PEI2k (MW: 2 kD) was reacted with 1-iodododecane as previously reported (8). The product was treated with NaOH and dialyzed against water to obtain N-alkyl-PEI2k upon freeze-drying. The product N-alkyl-PEI2k was dissolved in methanol and added dropwise to a DL-glycidol methanol solution (12). The mixture was stirred for 3 days and dialyzed in water, yielding N-alkyl-PEI2k-LAC.

Hydrophobic SPIO nanoparticles were synthesized using the methods described by Sun et al. (13). After drying under argon gas, SPIO nanoparticles were redispersed in chloroform and mixed with N-alkyl-PEI2k-LAC at a mass ratio of 1:0.6. The mixture was transferred to water under sonication and shaken for 24 h, followed by rotary evaporation to obtain chloroform-free N-alkyl-PEI2k-LAC/SPIO nanocomposites.

### Characterization of N-alkyl-PEI2k-LAC/SPIO Nanocomposites

Water-soluble N-alkyl-PEI2k-LAC/SPIO nanocomposites were characterized using dynamic light scattering (DLS) and electron microscopy (SEM). *T*_2_ relaxation times were measured with a 1.5 T MR system (Siemens Sonata) at room temperature. Briefly, the Fe concentration of the nanocomposites in water was detected using atomic absorption spectrometry; then, gelatin phantoms containing gradient Fe concentrations of the nanocomposite were prepared. The T_2_ relaxation time was detected using MR imaging with a spin echo sequence (TR of 5,000 ms, TE ranging from 6 to 500 ms). The relaxivity value (*r*_2_) was calculated from a curve of 1/T2 relaxation time (s^−1^) *vs.* the iron concentration (mM Fe) (12).

### Characterization of the N-alkyl-PEI2k-LAC/SPIO/siRNA Complex

According to the target gene sequence selection methods described in literature (14), an siRNA targeting the *IDO* mRNA was synthesized by Ruibo Biological Co., Ltd. The antisense strand is 5′GUUCUAGAAGGAUCCUUGA3′. Alkyl-PEI2K-LAC/SPIO was mixed with the siRNA in PBS at different N:P molar ratios (0, 2.5, 5, 7.5, 10, 12.5, and 15, respectively). The complexes were subjected to gel electrophoresis to detect the optimal binding concentration. Then, alkyl-PEI2k-LAC/SPIO and siRNA were mixed at an N:P ratio of five for 20 min. A gradient mass of heparin or serum was separately added to the mixture to test siRNA-release capacity and stability of the complex in serum.

### Preparation of Cells and siRNA-Transfected Dendritic Cell Vaccines

The generation of murine DCs has been described in our previous report (12). Briefly, the bone marrow of Balb/C female mice was washed into RPMI-1640 medium and centrifuged to collect the cell pellet. The cells were cultured in flasks and stimulated with GM-CSF (20 ng/ml). On day 8, the non-adherent population was harvested. LPS (100 ng/ml, Sigma-Aldrich) and TNF-α (20 ng/ml, Peprotech) were added and cells were cultured for another 2 days to obtain mature DCs. The purity of DCs was determined by measuring CD11c expression using fluorescence activated cell sorting (FACS).

The 4T1 cells were cultured in RPMI-1640 medium containing 10% fetal bovine serum and 5% penicillin–streptomycin at 37°C in the presence of 5% CO_2_. Approximately 3 days later, the cells in the logarithmic growth phase were sub-cultured.

Using preparation methods described in the literature (15), the cultured 4T1 breast cancer cells were lysed by subjecting them to six freeze–thaw cycles and a 30 min incubation in a water bath with ultrasound exposure before being passed over a 0.2 μm filter on the benchtop to collect the filtrate. On day 8 of culture, DCs were co-incubated with the N-alkyl-PEI2k-LAC/SPIO/siRNA complexes at 6 μg Fe/ml for 6 h; the 4T1 lysate was added at a ratio of 3:1 (4T1 cells:DCs) and incubated for 48 h, and then cells were treated with LPS (100 ng/ml) for 12 h before harvest to promote maturation.

### *In Vitro* Transfection of N-Alkyl-PEI2k-LAC/SPIO/siRNA

FITC-conjugated N-alkyl-PEI2k-LAC/SPIO was mixed with the siRNA at an N:P ratio of five for 20 min to determine if the complexes would be successfully engulfed by the cells. The complexes were collected and added to the culture medium of immature DCs at 6 μg Fe/ml. After a 6 h incubation, cells were collected and fixed, incubated with DAPI for 15 min, and then washed with PBS. Fifty microliters of the cell suspension were dropped on a slide for confocal laser scanning microscopy (CLSM). For the analysis of the transfection efficiency, immature DCs were plated in a 48-well plate at a density of 2 × 10^5^ cells/ml; then, N-alkyl-PEI2k-LAC/SPIO/siRNA (n = 5) was added at Fe concentrations of 0, 2, 4, 6, 8, 10, and 12 μg/ml. After a 6 h incubation the plates were cooled to room temperature, the iron reaction reagent was added, and the absorbance was read at 570 nm using a plate reader (Varioskan Flash, Thermo Scientific). The N-alkyl-PEI2k-LAC/SPIO/siRNA complexes were added to immature DCs at 6 μg Fe/ml to determine potential unwanted effects on DCs. Six hours later, the medium was replaced with three volumes of 4T1 breast tumor cell lysate, as described in detail below. After a 48 h incubation, DCVs transfected with N-alkyl-PEI2k-LAC/SPIO/siRNA complexes were obtained. The siRNA-laden DCVs were collected and transferred to a 1.5 ml centrifuge tube, with siRNA-free and immature DCs and siRNA-free DCVs as controls, and then incubated with antibodies to detect the expression of the surface molecular markers MHC-II, CD11c, CD80, CD86, and CCR7 using FACS.

### Western Blotting

On day 8 of culture, DCs were divided into two groups, one was incubated with IFN-*γ* (200 U/ml), LPS (100 ng/ml) and TNF-α (20 ng/ml), and the other was treated only with LPS (100 ng/ml) and TNF-α (20 ng/ml). Forty-eight hours later, the cells were collected for Western blotting to analyze the expression of IDO (16).

### Dendritic Cell Vaccine Treatment Protocols for 4T1 Mice and *In Vivo* Magnetic Resonance Imaging

After hair removal, the mammary fat pads of 6-week-old Balb/C female mice were injected with 1 × 10^6^ 4T1 cells. Vaccines were injected using previously reported procedures (17). Briefly, TNF-α (100 ng/ml) was subcutaneously injected into the left posterior footpad of the mice to pre-sensitize the local tissues. After 48 h, PBS, DC, DCV, and *IDO* siRNA-laden DCV were injected at the same sites, respectively. About 100 µl of PBS, 3 × 10^6^ cells/ft of DC, or 3 × 10^6^ cells/ft DCV were injected on the day of tumor inoculation and thereafter repeated once a week for two times. Mice with breast cancer were randomly grouped into four (n = 5 mice per group). The following treatment regimens were planned: (1) PBS injected into the left footpad; (2) N-alkyl-PEI2k-LAC/SPIO DCs, activated by LPS (100 ng/ml) and TNF-α (20 ng/ml), injected into the left footpad; (3) N-alkyl-PEI2k-LAC/SPIO DCVs injected into the left footpad; and (4) *IDO* siRNA-laden DCVs injected into the left footpad. The mice were imaged under a Philips 3.0 T MR scanner using a 25 mm small animal coil at 24 h before and 48 h after each injection. Echoes of popliteal lymph nodes (LNs) were measured primarily using a *T*_2_-wighted fast spin echo (TSE) sequence (TR = 2300 ms, TE = 121 ms, matrix = 148 × 148, slice thickness = 0.6/0.0 mm, FOV = 30 mm, flip angle = 90°, NSA = 10).

### Evaluation of the Therapeutic Effects of Indoleamine 2, 3-Dioxygenase siRNA-Laden Dendritic Cell Vaccines on Tumors

After the inoculation of 4T1 cells, the tumor sizes were measured on day 3 and repeated every 3 days thereafter for 4 repeats. The tumor volume *S* was calculated using the formula, *S = 0.5 × a^2^ × b*, where *a* represents the short diameter of the tumor and *b* represents the long diameter of the tumor (18).

### Statistical Analysis

SPSS 13.0 software was employed for statistical analyses. Independent sample T-test was used to test the statistical significance of differences between two groups, and one-way analysis of variance was used to determine the statistical significance of differences among more groups. *P* < 0.05 indicates a significant difference.

## Results

### Characterization of the Nanocomposites

**Figure 2:**
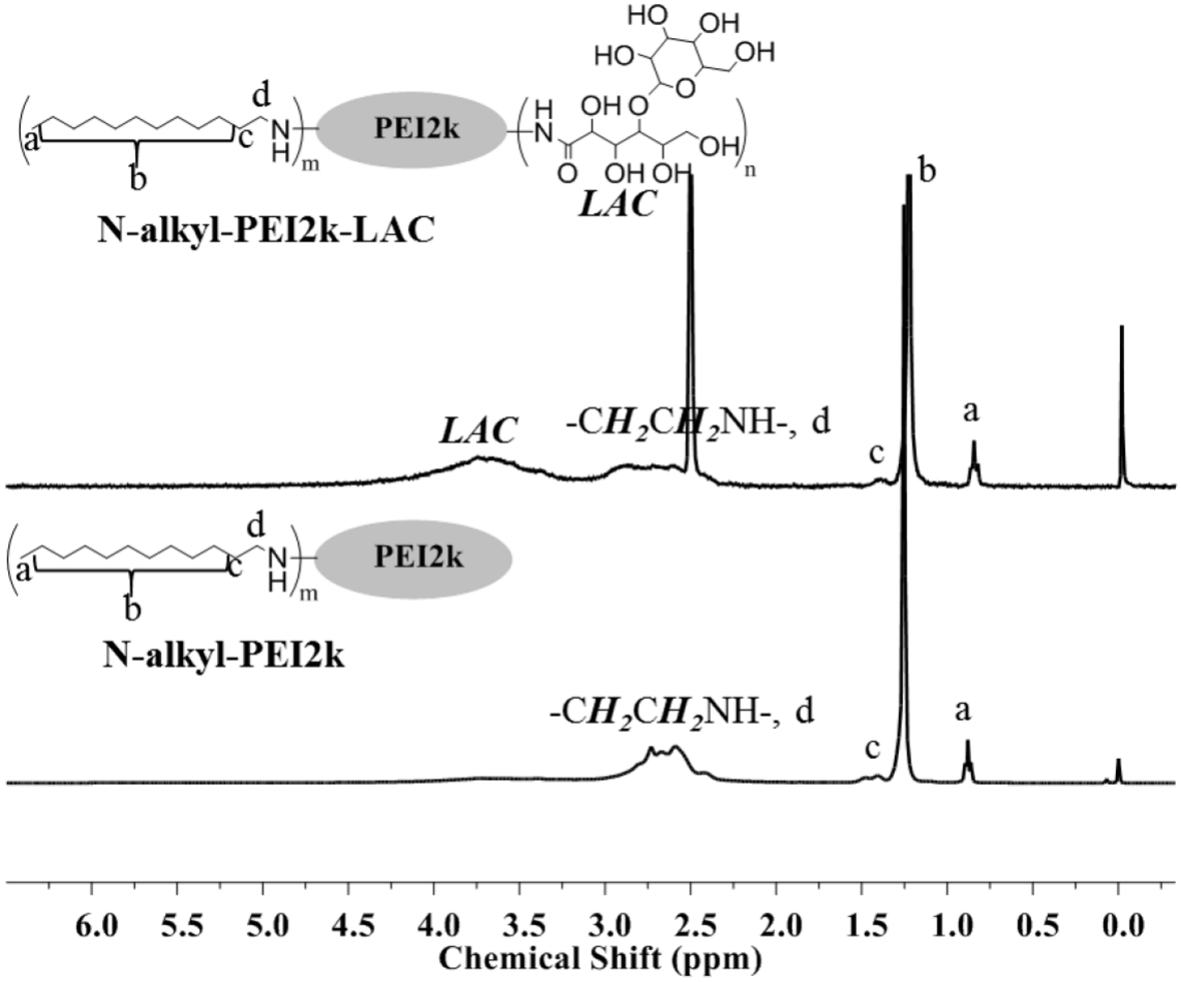
^1^H-NMR spectra of N-alkyl-PEI2k-LAC (DMSO) and N-alkyl-PEI2k (CDCl3).

The chemical structures of amphiphilic N-alkyl-PEI2k-LAC and N-alkyl-PEI2k were characterized using ^1^H NMR and elemental analysis (**Figure 2**). N-alkyl-PEI2k: ^1^H NMR (400 MHz, CDCl_3_) *δ* 3.15–2.21 (m, −CH_2_CH_2_NH−, −CH_2_CH_2_ (CH_2_)_9_CH_3_), 1.41 (br, −CH_2_CH_2_(CH_2_)_9_CH_3_), 1.25 (s, −CH_2_CH_2_(CH_2_)_9_CH_3_), 0.88 (t, −CH_2_(CH_2_)_10_CH_3_); Elemental analysis: C, 56.489%; N, 19.07%; H, 9.209%. N-alkyl-PEI2k-LAC: ^1^H NMR (300 MHz, DMSO) *δ* 4.55–3.21 (m, LAC), 3.19–2.25 (m, −CH_2_CH_2_NH−, −CH_2_CH_2_(CH_2_)_9_CH_3_), 1.39 (br, −CH_2_CH_2_(CH_2_)_9_CH_3_), 1.22 (s, −CH_2_CH_2_(CH_2_)_9_CH_3_), 0.84 (s, −CH_2_(CH_2_)_10_CH_3_). Elemental analysis: C, 46.188; N, 11.499%; H, 10.223%. Grafting ratio of alkyl and lactose were separately 12.1 and 10.3%, calculated upon elemental analysis results.

**Figure 3:**
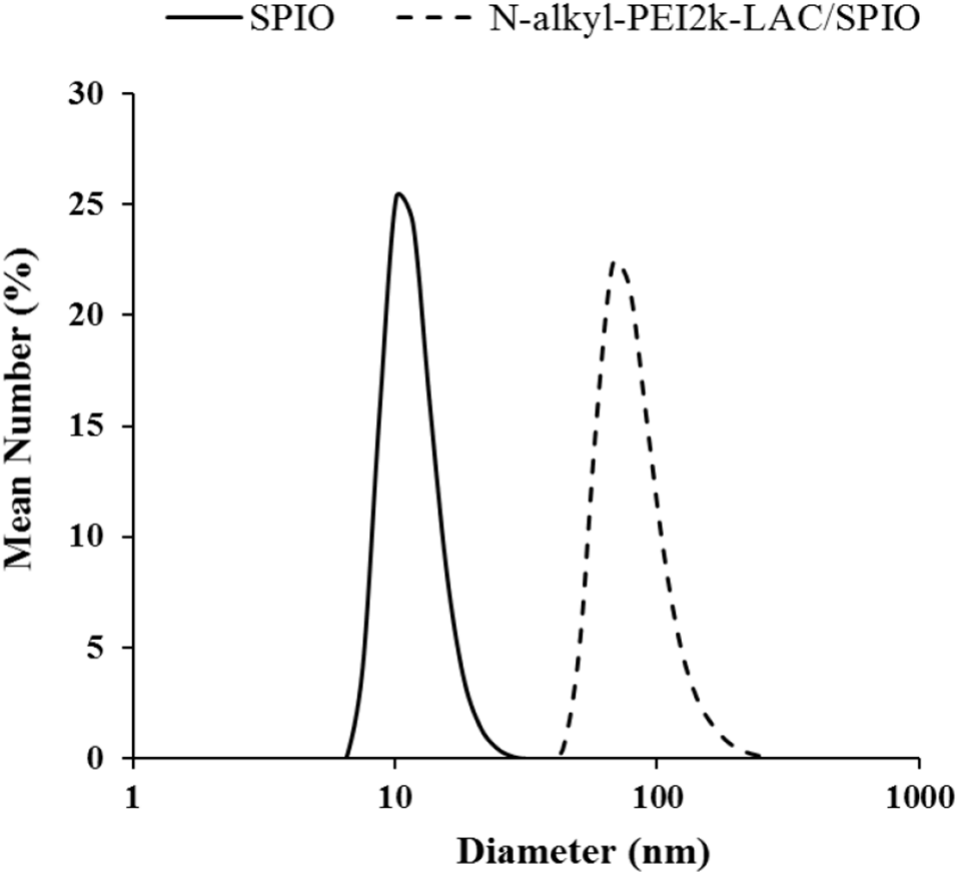
DLS of SPIO nanocrystals in hexane and N-alkyl-PEI2k-LAC/SPIO nanocomposites.

**Figure 4:**
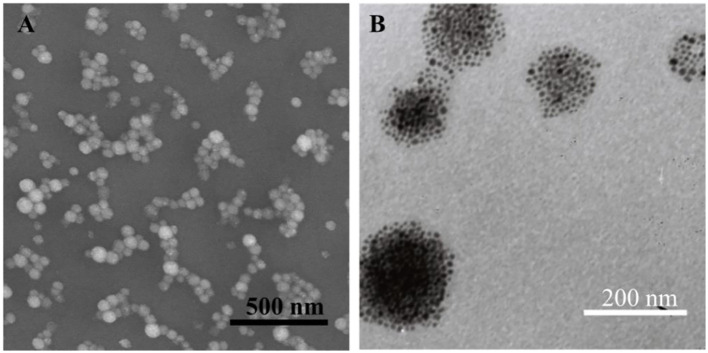
SEM **(A)** and TEM **(B)** images of N-alkyl-PEI2k-LAC/SPIO nanocomposites.

The monodisperse SPIO nanocrystals and amphiphilic material (mass ratio: N-alkyl-PEI2k-LAC : SPIO = 0.6) were used to form N-alkyl-PEI2k-LAC/SPIO nanocomposites in the water phase. The nanocomposites dispersed in water stably, with a diameter of 83.0 ± 26.5 nm obtained using DLS (**Figure 3**). The dry sample presented as spherical particles in SEM images, and SPIO nanocrystals aggregated into nanoclusters in the composites in TEM images (**Figure 4**). The surface charge of the SPIO nanocomposites remained positive with a zeta potential of 34.6 ± 1.1 mV, a value that is lower than N-alkyl-PEI2k/SPIO nanocomposites (approximately 40 mV), due to lactose partially shielding the positive electric charge on PEI.

**Figure 5:**
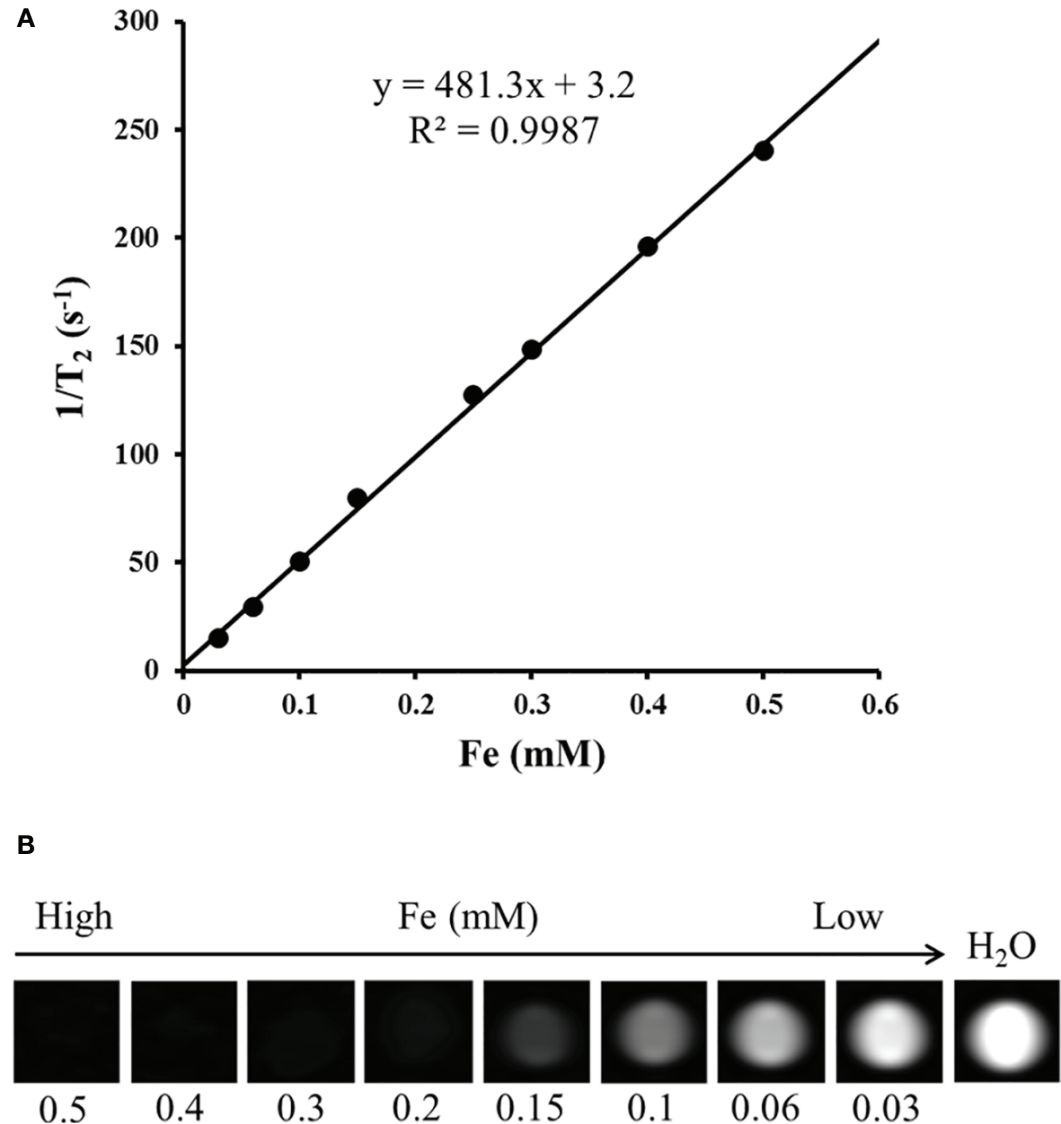
*T*_2_ relaxation rate as a function of the Fe concentration for N-alkyl-PEI2k-LAC/SPIO nanocomposites at 1.5 T **(A)**; *T*_2_-weighted MR images of N-alkyl-PEI2k-LAC/SPIO nanocomposites in water **(B)**.

Gelatin phantoms containing gradient Fe concentrations of the nanocomposite were detected using spin echo *T*_2_WI at different echo times with a clinical 1.5 T MR imager at room temperature to measure the *T*_2_ relaxivitiy of the nanocomposites. Signal intensities (SIs) were acquired to calculate the *T*_2_ relaxivitiy (r_2_) of the nanocomposites of 481.3 mM^−1^s^−1^ (**Figure 5**).

### Characterization of the N-alkyl-PEI2k-LAC/SPIO/siRNA Complex

**Figure 6:**
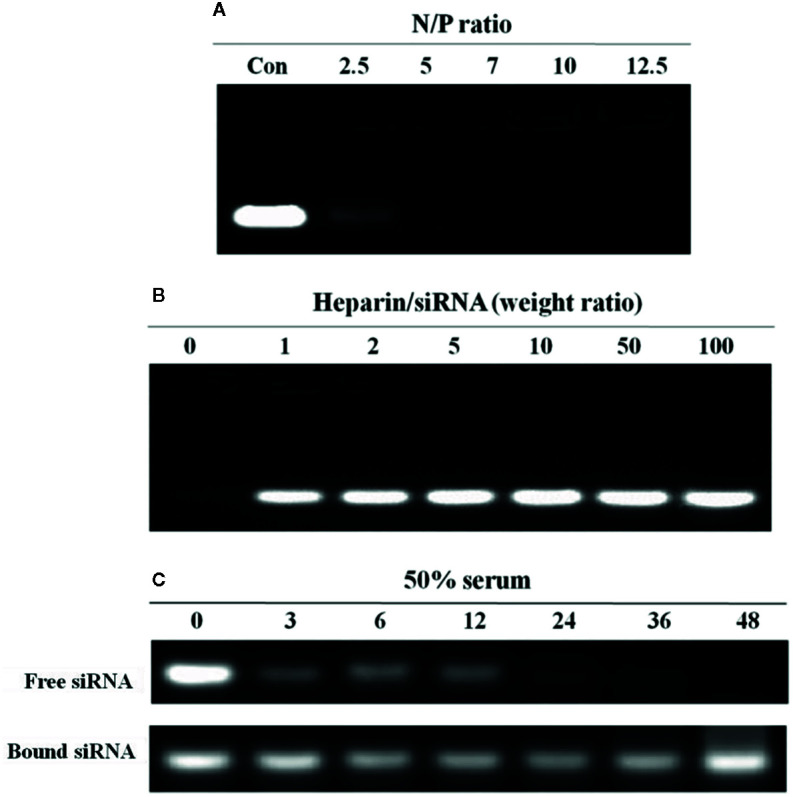
Agarose gel electrophoresis analysis of the N-alkyl-PEI2k-LAC/SPIO/siRNA complexes. **(A)** At an N:P ratio ≥ 5, the nanocomposites completely bound the siRNA. **(B)** At a heparin/siRNA mass ratio ≥ 5, a substantial amount of the siRNA was released. **(C)** Serum stability of the siRNA bound to or free from N-alkyl-PEI2k-LAC/SPIO. The free siRNA or N-alkyl-PEI2k-LAC/SPIO/siRNA complexes were incubated with 50% serum for the indicated times. Heparin was used to release the bound siRNA from the nanocomposites.

N-alkyl-PEI2k-LAC/SPIO was incubated with the siRNA at different N:P molar ratios and then subjected to gel electrophoresis. With the increase in the N:P ratio, siRNA electromigration was increasingly blocked, and electromigration was completely blocked at an N:P ratio ≥ 5. Based on these results, the siRNA was successfully bound to the surface of the nanocomposites (**Figure 6A**).

In a heparin decomplexation assay, N-alkyl-PEI2k-LAC/SPIO/siRNA complexes (N:P = 5) were loaded on agarose gel. The electrophoresis assay showed that the siRNA was released from the complexes when heparin was added. At a heparin:siRNA mass ratio ≥ 5, the released siRNA tended to be stable and its concentration no longer increased (**Figure 6B**).

In a serum stability assay, N-alkyl-PEI2k-LAC/SPIO/siRNA complexes (N:P = 5) and the unbound siRNA were separately incubated with 50% fetal bovine serum (FBS) for different periods. As a result, the siRNA in the complexes was substantially more stable in serum than the unbound siRNA; and even after a 24 h incubation, the siRNA still stably resided on the nanocomposite surface without observable degradation (**Figure 6C**).

### *In Vitro* Transfection of N-alkyl-PEI2k-LAC/SPIO/siRNA

**Figure 7:**
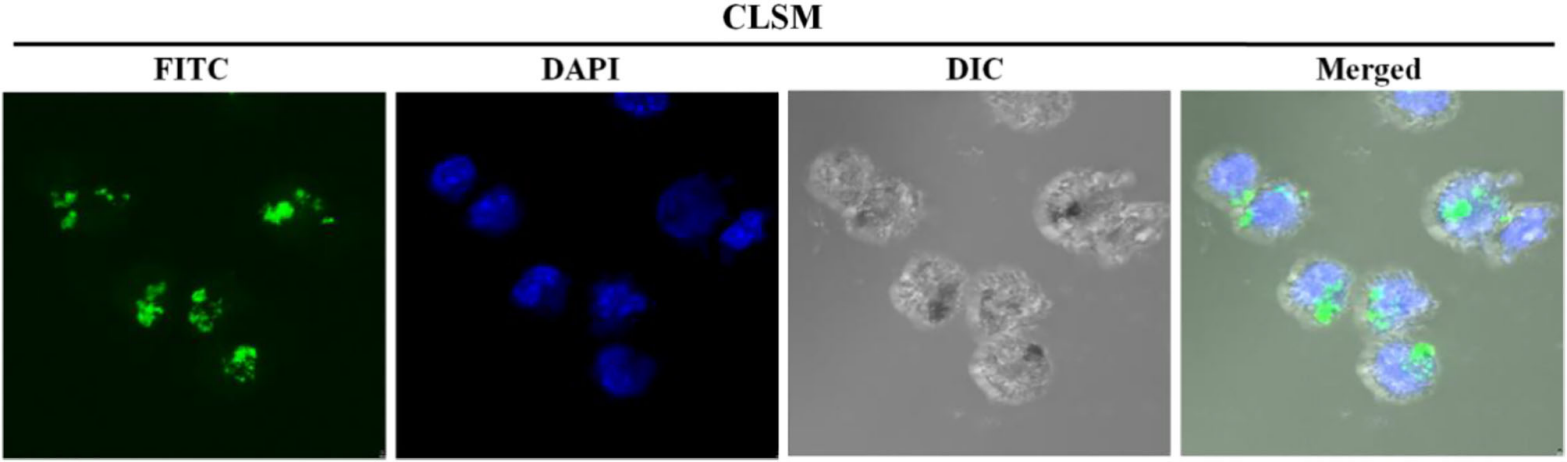
CLSM images of immature siRNA-laden DC showing clusters of green fluorescent particles around the DAPI blue-stained nuclei. DIC images display the pseudopodia of the cells.

FITC-conjugated N-alkyl-PEI2k-LAC/SPIO was mixed with the siRNA; then, the complexes were transfected into DCs by coincubation. CLSM revealed clusters of green fluorescent particles around the blue-stained nuclei. Differential interference contrast (DIC) imaging further showed pseudopodia of the cells, suggesting that they are siRNA-laden DCs (**Figure 7**).

**Figure 8:**
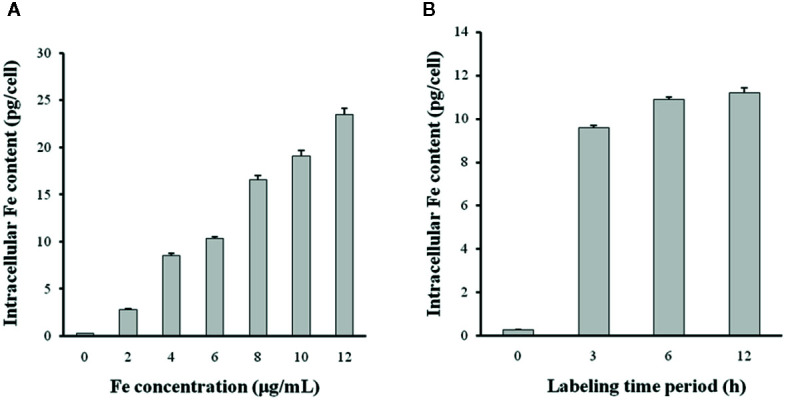
The siRNA transfection efficiency was altered by the N-alkyl-PEI2k-LAC/SPIO/siRNA concentration **(A)** and transfection time period **(B)**. Higher concentrations of the complexes or a longer transfection time period increases the Fe content within DCs (n = 5).

The intracellular Fe content was measured using a colorimetric ferrozine assay. DCs took up the N-alkyl-PEI2k-LAC/SPIO/siRNA complexes in a time- and dose-dependent manner (**Figure 8**). After a 6 h incubation, the intracellular Fe content tended to be stable, indicating that at least 6 h is necessary for sufficient transfection. A slightly lower concentration of the N-alkyl-PEI2k-LAC/SPIO/siRNA complexes, 6 μg Fe/ml, was adopted for subsequent *in vivo* studies to ensure ample antigen loading.

**Figure 9:**
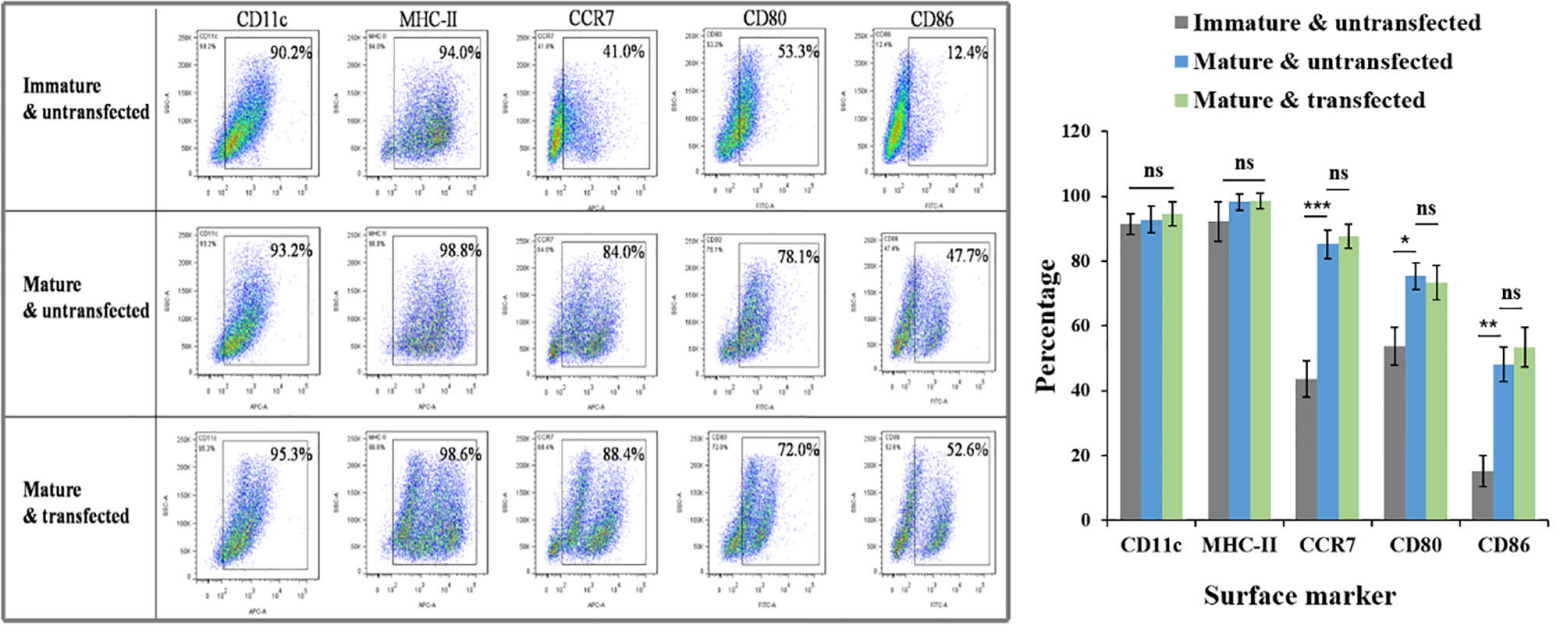
Effects of N-alkyl-PEI2k-LAC/SPIO/siRNA transfection on the maturation phenotype and marker expression of DCs. FACS assay showed that siRNA-laden mature DCs expressed similar levels in biomarkers MHC-II, CD80, CD86, and CCR7 to siRNA-free mature DCs, but significantly higher levels than immature DCs in CCR7, CD80 and CD86. A two-tailed unpaired Student’s t test was performed for two-group comparisons, and the results with significant difference were marked in * *P* < 0.05, **P < 0.01, or *** *P* < 0.001, respectively. ns, no significance.

The surface phenotype of DCs was detected using a FACS assay to probe possible adverse effects of the transfection process on DC maturation. CD11c is a marker of the purity of DCs. Our culture produced approximately 90% CD11c positive cells. Four other biomarkers, MHC-II, CD80, CD86, and CCR7, showed similar expression on siRNA-laden mature DCs to siRNA-free mature DCs, but the expression of CD80, CD86, and CCR7 was significantly higher than on immature DCs (*P* < 0.05 for all of them), suggesting the excellent biosafety of transfection (**Figure 9**).

### Western Blot

**Figure 10:**
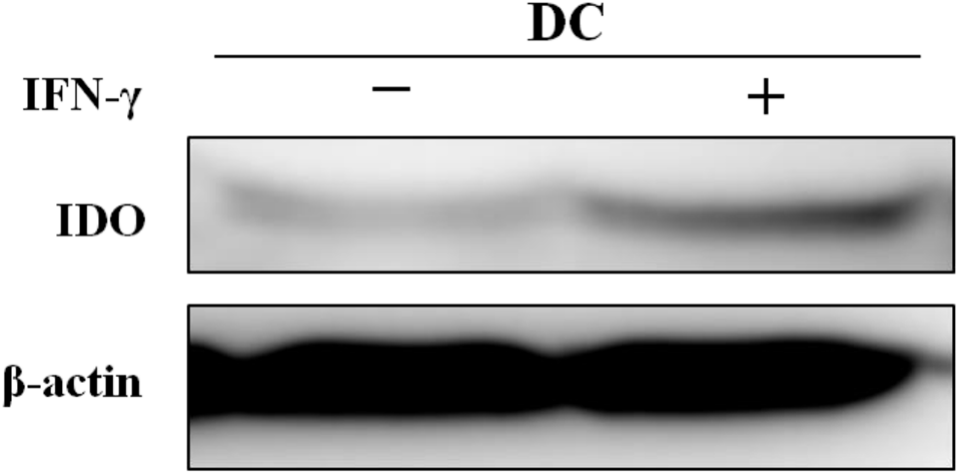
*In vitro* experiments showing the effects of IFN-*γ* on IDO expression in DCs.

Western blotting was performed on two groups of mature DCs treated with or without IFN-γ to analyze the expression of the target protein IDO in DCs. Replicate experiments revealed that IFN-*γ*-induced DCs expressed slightly higher levels of IDO (**Figure 10**).

### *In Vivo* Magnetic Resonance Imaging of Tumors Treated With *Indoleamine 2, 3-Dioxygenase* siRNA-Laden Dendritic Cell Vaccines

**Figure 11:**
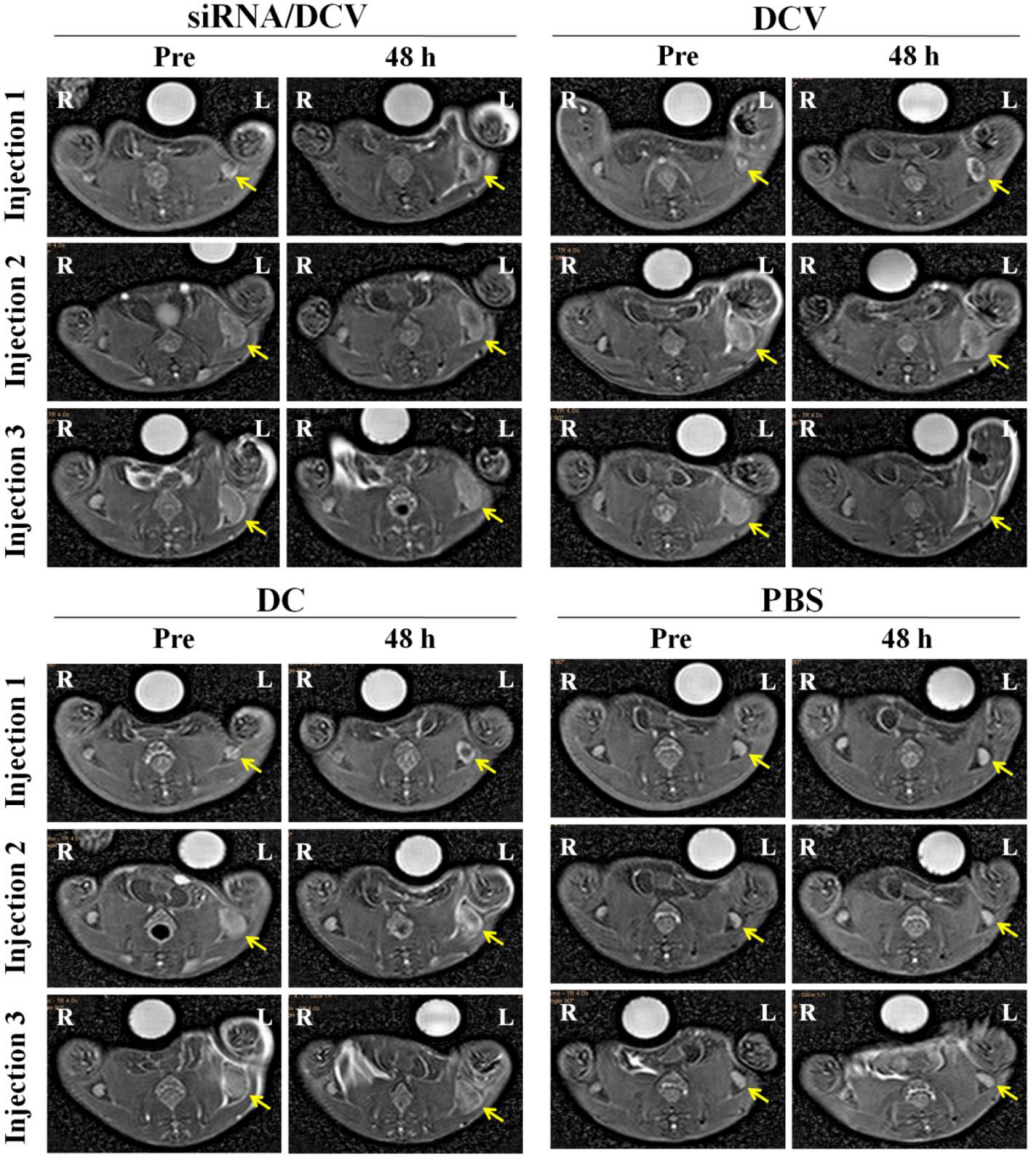
The left hind footpads were injected three times (1 injection/week) with the siRNA/DC vaccine (siRNA/DCVs), DC vaccine (DCVs), DC or PBS. At 24 h after each injection, MR images of all groups but the PBS group showed decreased signals within enlarged popliteal lymph nodes (yellow arrows).

MR imaging was performed to observe whether the injected vaccines migrated to the draining lymph nodes as expected and resulted in changes in SI. MR imaging of bilateral popliteal lymph nodes was performed on the four groups of tumor-bearing mice before and after each footpad injection. Vaccines were injected weekly for three injections, and MR images were correspondingly obtained at two time points, immediately before and 48 h after each injection. Left popliteal nodes of all DC groups were gradually enlarged. In particular, the nodes were strikingly enlarged 1 week after the first injection. On the TSE images captured 48 h after the first injection, the signal was clearly reduced within the nodes’ central zone of all DC groups; at 48 h after the second or third injections, a focal signal reduction was still visible, but not as clear as after the first injection (**Figure 11**).

### Evaluation of the Antitumor Therapeutic Effects

**Figure 12:**
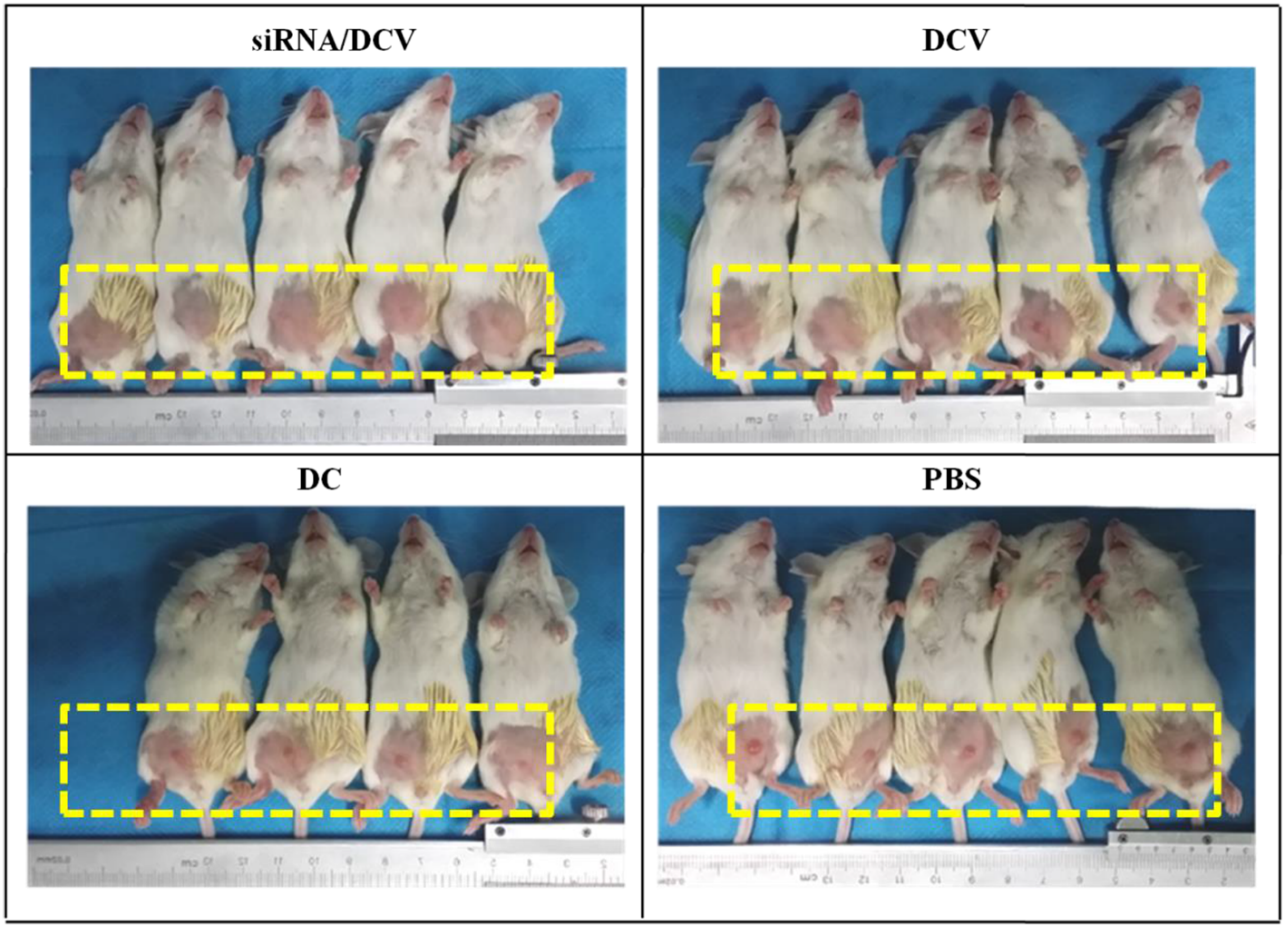
Photos taken on the 6^th^ day after tumor cell inoculation. Tumors grew fastest in the PBS group, followed by the DC group and the two vaccine groups.

**Figure 13:**
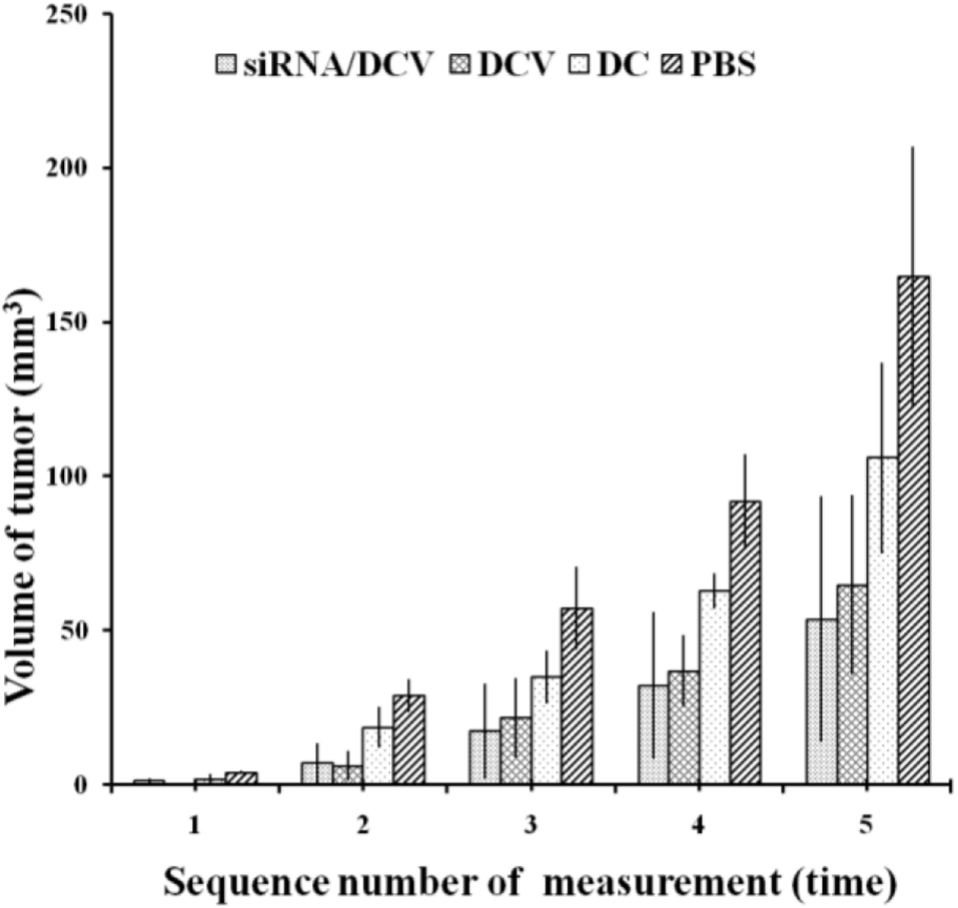
Tumor volumes were compared among the four groups. A significant difference was observed between the PBS group and the other groups (n = 5, *P* < 0.05). Moreover, measurements at the second to fifth time points showed that tumors in the siRNA/DCV group or the DCV group were both smaller than tumors in the DC group (n = 5, *P* < 0.05), suggesting the presence of a specific immune response.

By the end of the experiment, all mice survived, except for one mouse in the N-alkyl-PEI2k-LAC/SPIO DC group that died unexpectedly. After the inoculation of 4T1 tumor cells, tumor volume was measured and calculated once every 3 days for five measurements. Tumor sizes increased gradually, regardless of treatment with PBS, DC, DCVs or *IDO* siRNA-laden DCVs. Notably, tumors in the PBS group grew the fastest, followed by the tumors in the DC group. Tumors in the two vaccine groups grew at similar rates (**Figure 12**). The tumor volume was analyzed using ANOVA, and the *IDO* siRNA-laden DCV group had comparable tumor volumes to the DCV group, but significantly larger volumes than the PBS or the DC group (P < 0.05 for both) (**Figure 13**).

## Discussion

### N-alkyl-PEI2k-LAC/SPIO as Magnetic Resonance Imaging-Visible Gene Nanovector Showed Favorable Properties

This study utilizes lactobionic acid to superficially modify N-alkyl-PEI2k/SPIO. The generated nanocomposites possess favorable physicochemical and MR imaging properties, as confirmed by ^1^H NMR, DLS, SEM and MR imaging. We studied their capabilities of binding or releasing the siRNA, the serum stability of the siRNA in the complexes, gene transfection efficiency, and their effects on DCs to obtain a better understanding of their biological properties as nanocarriers for gene transfection. Similar to the previously reported N-alkyl-PEI2k/SPIO, at an N:P ratio ≥ 5, N-alkyl-PEI2k-LAC/SPIO almost completely bound the siRNA, suggesting that the lactose modification method has little effect on the ability of the nanocomplex to bind to siRNA (9, 19). At a heparin/siRNA mass ratio ≥ 1, the siRNA was fully released to silence the target genes. Moreover, a high concentration of the siRNA persisted in serum even at 48 after the interaction with N-alkyl-PEI2k-LAC/SPIO, indicating that the nanocomposites provide reliably protect the siRNA from enzymolysis *in vivo*. Both CLSM and intracellular Fe content measurements showed that the transfection of N-alkyl-PEI2k-LAC/SPIO/*IDO*-siRNA was successful and depended on both the time and concentration. The molecular phenotype of a cell, to some extent, represents its properties and functions. FACS results showed that expression of four important molecular markers (MHC-II, CD80, CD86, and CCR7) on DCs transfected with the siRNA complex at 6 μg Fe/ml was not distinctly different from siRNA-free mature DCs. In recent years, some authors have noted that nanoparticles often facilitated maturation of DCs (20, 21). In a previous study, we also found that glycoidol-modified alkyl-PEI/SPIO nanocomposites up-regulated expression of MHC-II, CD80, CD86, and CCR7 if DCs were labeled prior to maturation (12). When DCs are matured, they exhibit function reduction in capturing antigens and function enhancement in migration and antigen-presentation. We do not think the effects of nanocomposites to DCs are conducive because the non-specific stimulation may lead to impaired capture of the antigens (12). In current study, the effect was not so evident for N-alkyl-PEI2k-LAC/SPIO nanocomposites, as shown by the FACS results. The improved properties perhaps partly attribute to the lactose modification that reduced surface cations of the nanocomposites. DCs are the cells highly sensitive to external stimuli (12). The expression of their surface markers was not obviously affected in this study, suggesting the biological friendliness of the nanovector and the transfection process.

### In Vivo Homing of *Indoleamine 2, 3-Dioxygenase* siRNA-Laden Dendritic Cell Vaccines to Lymph Nodes Can Be Successfully Detected by Magnetic Resonance Imaging

During the treatment of 4T1 tumor-bearing mice with DCVs, MR imaging showed that *IDO* siRNA-laden DCVs that were injected into the murine left footpads successfully migrated to the draining lymph nodes after 48 h, resulting in nodal enlargement and darkening of the MR signal in the central zone. The central distribution indicates a decrease in the MR signal caused by active homing of the N-alkyl-PEI2k-LAC/SPIO nanocomposite containing DCVs rather than the nanocomposites themselves that are present mainly in the peripheral distribution (22). The tumor treatment adopted in this study mainly referred to the methodology reported by Hegmans JP (17). In the present study, regardless of the material that was injected, the tumors of the mice grew over time. However, the tumors of the PBS group grew fastest, followed by the DC group, indicating that both DCVs and *IDO* siRNA-laden DCVs actively inhibit tumor growth.

### Limitations of This Study and Possible Causes

Notably, in contrast to previous reports, DCVs transfected with or without *IDO*-siRNA produced similar levels of tumor inhibition in the present study. No significant difference in tumor size was observed between the two groups (23, 24). This lack of a difference in the tumor size is a major drawback of the study, as tumor is presumed to be regulated by the low expression of IDO in DCs, as verified by our repeated experiments. Typically, IFN-*γ* induction alone is sufficient to up-regulate IDO expression in DCs, but accumulating evidence showed that the up-regulation of IDO in DCs required ‘two signals’, *i.e.*, IFN-*γ* stimulation followed a second stimulus (such as IL-10, CD40 or LPS) (25, 26). In the present study, DCs underwent a two-stage stimulation with IFN-*γ* and LPS, but to our surprise, the expression of IDO in DCs remained low. We have not yet determined a rational explanation for these outcomes but presume that they may be associated with the factors listed below. a) The subtype of DCs. The DCs cultured in this study were mainly cDCs, not pDCs, which may partially explain the low expression of IDO. b) The method used for DC culture. We cultured DCs mainly using the methodology reported by Lutz MB (27). Vaccines prepared using the method described by Lutz MB have been reported to exert the best anticancer effect (17). A good therapeutic effect may require IDO low expression. c) The microenvironment in which DCs reside. The DCs cultured in the present study were obtained from normal tumor-free mice. However, tumor-mediated modulation of the humoral immune environment has been reported to contribute to the up-regulation of IDO in DCs (28). Nevertheless, the main purpose of this study was to show that an *IDO* siRNA could be successfully introduced into DCs with lab-made gene nanovectors and used as *in vivo* imaging tracer. This purpose has been achieved. Moreover, our research group has previously used vectors in an analogous molecular skeleton to repeatedly confirm that the transfection of an siRNA with these vectors effectively silences the target genes in cells (9, 19). In subsequent studies, we plan to select more suitable targets such as PD-L, A20 and DIgR2 to verify the validity of siRNA-mediated silencing.

## Conclusions

In this study we used, with success, the lactoslyated nanocomposites N-alkyl-PEI2k-LAC/SPIO as an MRI-visible gene nanovector for *IDO* siRNA transfection into DCs. After the *IDO* siRNA-laden DCVs were adoptively transferred to the mice with 4T1 tumors, *in vivo* MR imaging clearly showed them homing into the draining lymph nodes. In summary, N-alkyl-PEI2k-LAC/SPIO, a gene delivery system, is able to transfect nucleic acid fragments and track cell migration *in vivo* using MR imaging.

## Data Availability Statement

The raw data supporting the conclusions of this article will be made available by the authors, without undue reservation.

## Ethics Statement

All animal procedures were performed in accordance with the Guidelines for Care and Use of Laboratory Animals of North Sichuan Medical College, and experiments were approved by the Animal Ethics Committee of North Sichuan Medical College (P20191226).

## Author Contributions

HA, as the consultant, was responsible for the guiding of the experimental research. YX took charge of the overall design of the project as well as the quality and schedule control. CW prepared and tested the MRI nanocomposites, and CW, WZ and RJ implemented the biological experiments. All authors contributed to the article and approved the submitted version.

## Funding

This work was funded by the National Natural Science Foundation of China (81601490), Chongqing Science and Technology Foundation, and Chongqing Science and Technology Commission (cstc2018jscx-msybX0069 and cstc2016 shmszx130009).

## Conflict of Interest

The authors declare that the research was conducted in the absence of any commercial or financial relationships that could be construed as a potential conflict of interest.
